# Supplementary material for: Prevalence of Toxoplasma gondii in Galapagos birds: Inference of risk factors associated with diet
Source: PLoS One. 2023 Jul 5;18(7):e0287403. doi: 10.1371/journal.pone.0287403 (PMC10321649; doi:10.1371/journal.pone.0287403)
Supplement: Attachment — Submitted filename: Response to Reviewers.docx [file pone.0287403.s002.docx]

**Response to Reviewers**

Dear Editors and Reviewers,

Thank you very much for your comments and observations on the manuscript.

Here you will find the responses to each point raised:

Journal Requirements:

1. We have ensured that the manuscript meets PLOS ONE's style requirements.
2. We have provided additional information regarding the handling of the animals and the efforts to alleviate suffering.
3. We have included the statement “The funders had no role in study design, data collection and analysis, decision to publish, or preparation of the manuscript.” in the cover letter.
4. We have removed the funding-related text from the manuscript and would like to remain with the original Funding Statement since the people mentioned are part of The Galápagos Science Center-USFQ/University of North Carolina-Chapel Hill.
5. We have uploaded the study’s minimal underlying data set as Supporting Information.
6. We remade Figure 1 using QGIS and the base map OSM Standard from OpenStreeMap. We have included the required text that provides credit to OpenStreetMap by displaying their copyright notice following their attribution guidelines.
7. We have reviewed the reference list to ensure that it is complete and correct.

Reviewers’ comments:

- We have addressed the reviewers’ minor comments and are marked as track changes in the revised version of the article.
- We have corrected the storage conditions of the samples (all samples were stored at -20°C, -80C was written by mistake).
- The recording of the physical conditions, age and sex of the seabirds is no longer mentioned in the article since it was not used in our study.
- We have reconstructed the sentence in line 255.
- We have addressed the difference between Toxoplasmosis and *T. gondii* infection.
